# Gambling Dual Disorder: A Dual Disorder and Clinical Neuroscience Perspective

**DOI:** 10.3389/fpsyt.2020.589155

**Published:** 2020-11-24

**Authors:** Nestor Szerman, Francisco Ferre, Ignacio Basurte-Villamor, Pablo Vega, Beatriz Mesias, Rodrigo Marín-Navarrete, Celso Arango

**Affiliations:** ^1^WADD WPA Section Dual Disorders, Institute of Psychiatry and Mental Health Hospital General Universitario Gregorio Marañón, Madrid, Spain; ^2^Institute of Psychiatry and Mental Health Hospital General Universitario Gregorio Marañón, Madrid, Spain; ^3^Institute of Addictions, Madrid Salud, Madrid, Spain; ^4^National Institute of Psychiatry Ramón de la Fuente, Mexico City, Mexico; ^5^Institute of Psychiatry and Mental Health Hospital General Universitario Gregorio Marañón, IiSGM, CIBERSAM, School of Medicine, Universidad Complutense, Madrid, Spain

**Keywords:** Gambling disorder, review, dual disorders, clinical neuroscience, genetics, neuroscience, precision psychiatry

## Abstract

Several behaviors, including compulsive gambling, have been considered non-substance-related addictive disorders. Categorical mental disorders (e.g., DSM-5) are usually accompanied by very different symptomatic expressions (affective, behavioral, cognitive, substance abuse, personality traits). When these mental disorders occur with addictive disorders, either concomitantly or sequentially over the life span, this clinical condition is called a dual disorder. Gambling disorder (GD) has been associated with other categorical psychiatric diagnoses: attention deficit hyperactivity disorder, depression, bipolar disorder, social anxiety, schizophrenia, substance use disorder, antisocial personality disorder; and dimensional symptoms including higher impulsivity, poorer emotional wellbeing, cognitive distortion, psychosis, deficient self-regulation, suicide, poorer family environment, and greater mental distress. We are calling this clinical condition Gambling Dual Disorder. From a clinical perspective, it is clear that Gambling Dual Disorder is not the exception but rather the expectation, and this holds true not just for GD, but also for other mental disorders including other addictions. Mental disorders are viewed as biological disorders that involve brain circuits that implicate specific domains of cognition, emotion, and behavior. This narrative review presents the state of the art with respect to GD in order to address current matters from a dual disorder, precision psychiatry, and clinical neuroscience perspective, rather than the more subjective approach of symptomatology and clinical presentation. This review also presents Gambling Dual Disorder as a brain and neurodevelopmental disorder, including from the perspectives of evolutionary psychiatry, genetics, impulsivity as an endophenotype, the self-medication hypothesis, and sexual biological differences. The wide vision of the disease advances a paradigm shift, highlighting how GD and dual disorders should be conceptualized, diagnosed, and treated. Rethinking GD as part of a dual disorder is crucial for its appropriate conceptualization from the perspective of clinical neuroscience and precision psychiatry.

## Introduction

From an evolutionary perspective, rewarding behaviors such as social interactions, play, and gambling activity have been strongly conserved in evolution, and they are essential for the development and survival of humankind (1). However, in vulnerable individuals, gambling is not recreational and becomes a gambling disorder (GD). According to Potenza et al. (2), gambling is defined as “an activity that involves placing something of value at risk in the hopes of gaining something of greater value.” Common forms of gambling include casino gambling (blackjack and slot machines, for instance), lotteries, and internet gambling (poker, sports betting) (2). According to the American Psychiatric Association classification (DSM-5) (3), GD (previously, pathological gambling) is a mental condition, and since 2013 it has been considered an addictive disorder, like substance use disorders (SUDs) (4). Both SUDs and GD are chronic brain disorders and are strongly influenced by genetic, neurobiological, and psychosocial factors (4, 5).

According to all the evidence, there are similarities between certain SUDs and GD. Recent research establishes that, in spite of the similarities, there are also very important differences between addictions to different substances or gambling, and they can be inferred from the “drug choice model,” precision psychiatry concept and the dual disorders perspective (6, 7).

To clarify, the simple act of gambling cannot be classified as a disorder. In order to classify gambling as a mental disorder, it is necessary to consider its negative impact on the main areas of an individual's life (8), in addition to specific symptoms such as preoccupation with gambling, escalating wagers (tolerance), repeated attempts to quit, withdrawal symptoms, gambling as an escape, lying about gambling, borrowing money, and loss of relationships, among others (3). In the scientific literature, gambling addiction, and problem gambling occur on a continuum, with the former located at the end of the scale, whereas the latter is a less problematic behavior that may not lead to severe difficulties in the individual's life (9, 10). Nonetheless, it is very important to remark that GD represents the first recognized behavioral addiction with empirical evidence for prevention, diagnosis, and treatment (3). In recent years, the classification of mental disorders has been a matter of great debate, as advances in neuroscience have revealed many neurobiological correlates of mental disorders (11), together with a strong correlation with cognitive behavioral and personality measures (12).

The National Institute of Mental Health advocates the use of Research Domain Criteria (RDoC) to research mental disorders as an attempt to create a new kind of taxonomy for mental disorders based on dimensions of observable behaviors and neurobiological measures (13). Mental disorders are viewed as biological disorders that involve brain circuits implicating specific domains of cognition, emotion, and behavior. Identifying brain phenotypes in GD currently presents a great challenge. Many studies report the co-occurrence of GD with SUDs and other mental disorders, mental symptoms, or dysfunctional personality traits (2, 14–16). When mental disorders or symptoms occur with addictive disorders, either concomitantly or sequentially over the life span, this clinical condition is called a dual disorder (7, 17, 18). From a categorical diagnosis perspective, if gambling/gaming disorders are a result of other mental disorders, they cannot be considered a bona fide addiction. From an epidemiological and clinical perspective this disorder is not simply an addiction, but a dual disorder. Furthermore, from this point of view it is clear that dual disorders are not an exception but the norm (19).

The objective of the present narrative review is to provide the state of the art with respect to GD in order to address current matters based on a dual disorder, precision psychiatry and clinical neuroscience perspective, rather than the more subjective approach of symptomatology and clinical presentation. This review will also present the Gambling Dual Disorder as a brain and neurodevelopmental disorder including the perspectives of evolutionary psychiatry, genetics, impulsivity as an endophenotype, the self-medication hypothesis, and sexual biological differences.

## Epidemiology of Gambling Dual Disorder

**Figure 1:**
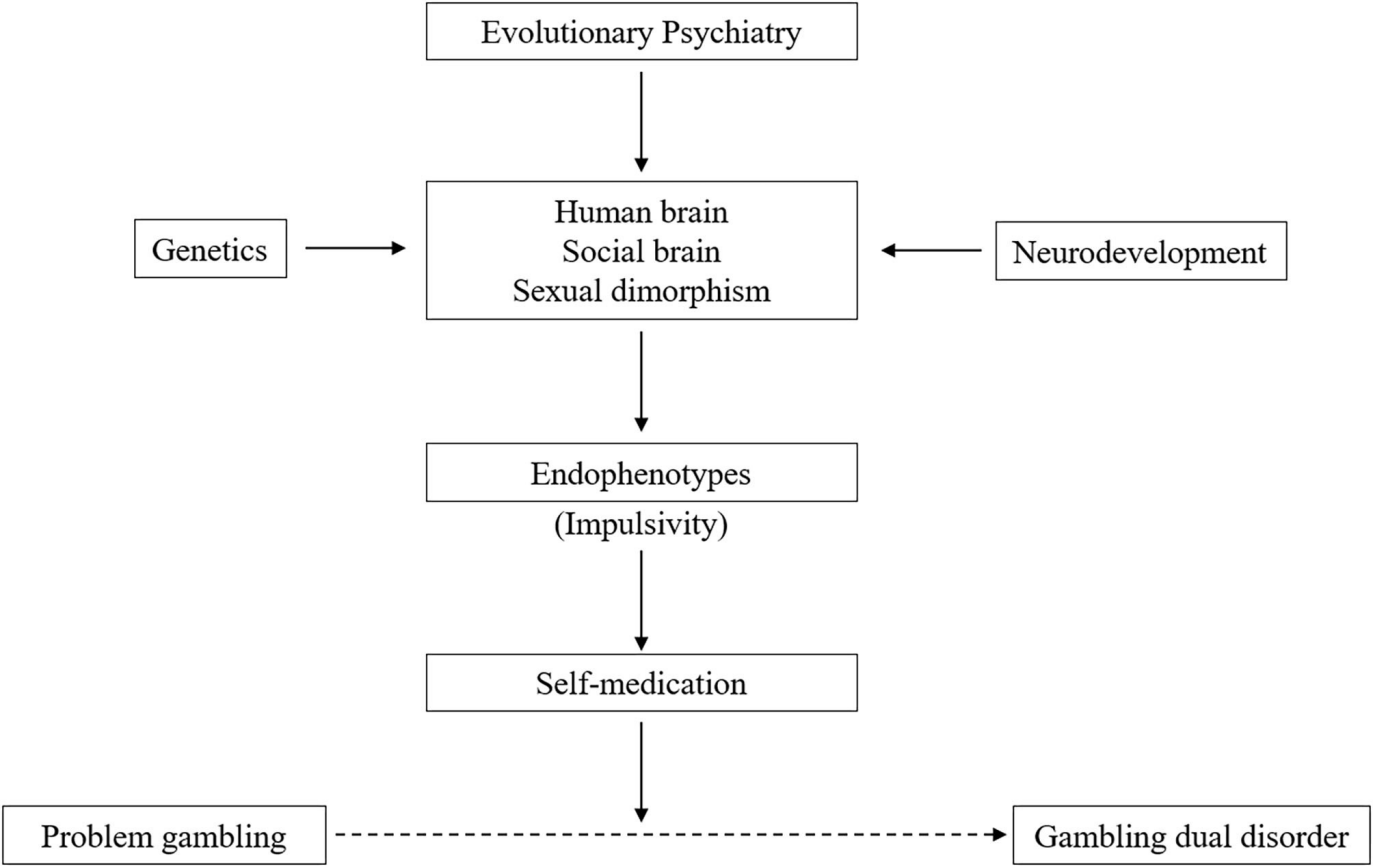
Conceptualization of gambling dual disorder.

A systematic review of general population studies highlighted the differences in GD prevalence across countries worldwide (0.1–5.8%) and Europe (0.1–3.4%) (20). Nevertheless, the lack of nationwide studies with representative samples made it difficult to directly compare prevalence rates. The prevalence rates also show ethnic differences, with rates more elevated in the Black population than other ethnics groups in the US (21, 22). A large National Epidemiologic Survey in the US found a higher rate of GD among Black (2.2%) and Native/Asian Americans (2.3%) compared with whites (1.2%) (23). Data from epidemiological studies should be interpreted cautiously because they have been affected by changes in the conceptualization and classification of GD in recent years. As in other epidemiological studies in psychiatry comparing prevalence rates among studies and countries, the variability of these results is associated with diverse factors, such as the instrument used for diagnosis or screening, the populations included, and the data collection method (face-to-face or telephone interviews, surveys, etc.) (2, 20). Similarly, studies carried out with clinical populations have reported higher prevalence rates (2). For instance, the prevalence of GD in psychiatric inpatients or individuals who have received treatment for SUDs is 6.9% and 4.3%, respectively. These findings corroborate the existence of the Gambling Dual Disorder. Indeed, according to the NESARC study, 96% of individuals with GD have one or more other psychiatric disorders and 64% have three or more (24, 25). It is relevant to note that this study did not consider personality disorders. SUDs, mood, impulse-control, and anxiety disorders, and other neuropsychiatric disorders (e.g., Parkinson's disease in 2.2–7%) are especially prevalent in individuals with GD (26, 27). Novel epidemiological studies evaluating internet gaming disorder have also confirmed the co-existence of other mental disorders, such as anxiety (92%), depression (89%), attention deficit hyperactivity disorder (ADHD, 85%), and social phobia with obsessive-compulsive symptoms (75%) (28). The concurrence of GD with substance use (tobacco, alcohol, or cocaine) is well established (29, 30). Indeed, up to 17% and 28% of individuals with GD report an illicit drug or alcohol use disorder, respectively (15). Despite extensive knowledge of mental disorders in individuals with GD, there is a lack of knowledge about the temporal sequencing between GD and other mental disorders (31). Finally, epidemiological findings highlight the difficulty in conceptualizing GD as a single nosological entity, defined only by gambling. Therefore, these findings support our proposal to coin the term Gambling Dual Disorder (Figure 1).

## Etiopathogenesis of Gambling Dual Disorder

Historically, etiological hypotheses associated with GD have evolved with advances in clinical neuroscience. They have changed from the premise of continuous exposure to pleasurable and reinforcing behaviors on the brain reward system (BRS) that produces neuroplastic changes and leads to the addictive behavior to explanations based on an alternative model, where the research, focused on compulsive drug use (or compulsive behavior), goes beyond simple reinforcement mechanisms on BRS (32). Research on dual disorders is going to identify vulnerable people, distinctive endophenotypes, and neurobehavioral and clinical traits predisposing individuals to the compulsive drug use (or gambling).

We are moving from models which use different phenomenological and symptomatic characteristics to define a GD, such as The Pathways Model (33), to a new perspective originated in clinical neuroscience and precision psychiatry, which incorporates genetics and neurobiology to explain an individual's vulnerability to developing a gambling dual disorder (7).

**Figure 2:**
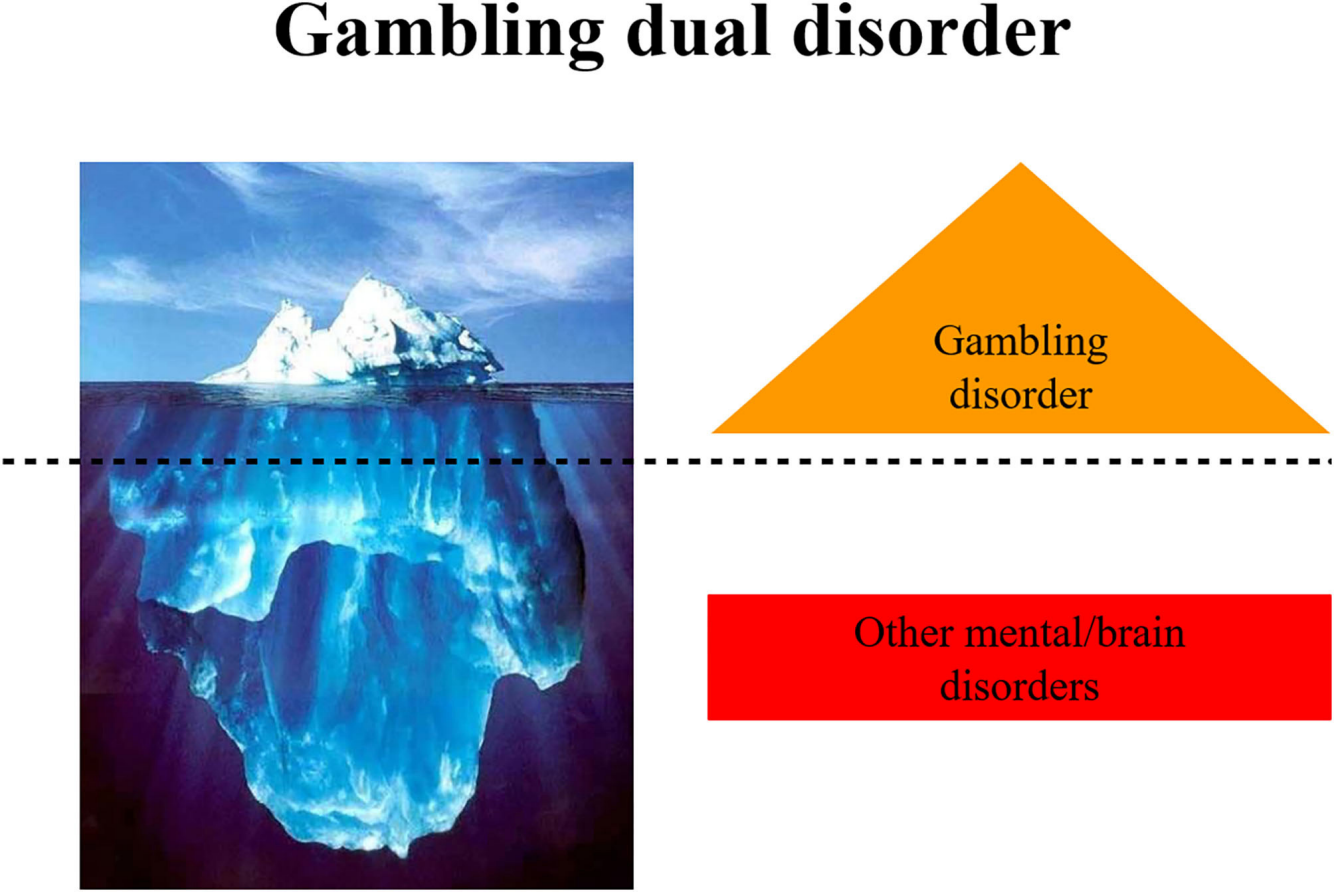
Scheme of the different perspectives that converge into gambling disorder.

In this sense, we address the perspective on Gambling Dual Disorder using evolutionary psychiatry, brain configuration, neurodevelopment, genetics, impulsivity as an endophenotype, the self-medication hypothesis, and sexual brain dimorphism (Figure 2).

### Evolutionary Psychiatry and Gambling Dual Disorder

From the evolutionary perspective, the human brain has developed circuits to cope with the vital objective of survival, such as searching for food and water, sex, breeding, and exploration of territory, but also skills for social interaction and recreational activity (e.g., gambling) (34, 35). All these behaviors are enjoyable and represent pleasurable experiences with a high reward value activating the brain's reward system. An important part of the evolutionary history of humankind is characterized by the development of a “social brain” that support a variety of emotional and cognitive mechanisms. These mechanisms can become dysfunctional, due to genetic and/or environmental factors, and lead to psychopathological changes (36). From an evolutionary perspective, “game-based gambling dates far back in human history as an almost universal activity” (37) and has undergone adaptive psychobiological changes for survival in ways similar to other behaviors (38). Addictive behaviors can be understood from the interaction of ancient evolutionary brain circuits and systems (designed to promote the search for natural rewards) and contemporary contexts (where potent drugs and gambling are easily available in our society) (39).

### Gambling Dual Disorder as a Brain Disorder

Mental functions, such as thinking, feeling, perceiving, decision making, self-control, and interacting, are based on the human brain (40). Notably, all rewarding behaviors needed for survival are based in the BRS. Similar to SUDs, GD is a consequence of the interaction between a genetic vulnerability and environmental factors, causing lasting adaptive changes in BRS function (34). Only a minority of individuals who experience pleasurable effects from games, gambling, food, sex, or shopping develop problematic behaviors or become addicted to them. Clinical neuroscience has revealed that addiction implies a set of interconnected processes that affect different mental functions, instead of being a disorder only or mainly defined by a unique behavior, such as the excessive and uncontrollable use of drugs (or gambling) (5). Decision making is an essential component of our daily life, and it becomes dysfunctional in a multitude of psychiatric conditions (impulse control disorder, psychosis, bipolar disorder, ADHD, and some addictions, including GD). A large brain network involving the BRS, prefrontal cortex (PFC), amygdala, and the nucleus accumbens is activated to achieve efficient decision making (41).

In relation to the debate on whether the BRS dopamine deficiency is a consequence of excessive gambling or if due to a pre-existing deficit of the dopaminergic/cortico-limbic system (i.e., BRS), from our alternative model, we propose that a dopaminergic deficit pre-exists in impulsive individuals and those with different severities of GD. On the contrary, non-impulsive people show resilience to developing addictive GD. In human studies, positive emotionality has been associated with dopamine D2 receptor availability (of healthy controls), and resilience to SUDs (42). Animal studies have demonstrated that impulsive rats exhibit lower levels of D2 receptors in the striatum than non-impulsive rats (43), and impulsivity is normalized in highly impulsive rats by prior exposure to cocaine (44). This evidence supports the possibility of the alternative proposal of a pre-existing deficit of the dopaminergic/cortico-limbic system. On the other hand, we do not have any evidence that the dopaminergic deficit of individuals with GD is a consequence of the continuous stimulation on the BRS.

In the human brain, the BRS is associated with fundamental biological systems (opioid, endocannabinoid, and nicotinic cholinergic system, among others), linked to survival as individuals and species, and also to mental functions, which, if disrupted, can give rise to different mental disorders (such as GD), and affect the voluntary control of behavior (45, 46). This can occur not only in substance addiction but also in other mental and behavioral disorders. Compulsive behaviors found in individuals with behavioral addictions, such as GD, may also originate from disruptions of the brain dopaminergic system (47). Individuals who are addicted to gambling show a clinical symptomatology that is correlated with neurobiological findings: less working memory and decision-making capacity, reduction in visual and auditory function, together with impairment of the prefrontal cortex (PFC) and deficiency of the BRS (48). Indeed in a systematic review, the authors have correlated the impaired activity in prefrontal cortex with a reduced cognitive control in GD patients (49). Another study also reported that individuals with GD had diminished volume in the left hippocampus and right amygdala, which in turn were associated with higher scores on the behavioral inhibition system scale, i.e., decreased tendency to avoid punishment (50). Another recent study has also highlighted the role of the cerebellum in GD (51). The strength of the functional connectivity in the cerebellar network was significantly correlated with severity of GD. A meta-analysis of functional magnetic resonance imaging studies revealed striatal hypoactivation in individuals with gambling addiction during reward anticipation, and during reward outcome in those with GD, compared with healthy controls (52). Studies have also correlated lower dopamine receptor availability in the striatum with mood-related impulsivity (53) and behavioral disinhibition (54, 55) in individuals with GD.

The GD is correlated with changes in frontal and limbic regions of the brain, similar to those found in individuals with an addiction to cocaine (56). Common and unique findings in GD and cocaine addiction, with respect to anticipatory reward and near-miss loss processing, suggest both shared and unique neurobiological elements (57). Furthermore, findings illustrate both similarities and differences in the neural correlates of drug cravings in cocaine addiction and gambling urges in GD (58).

There is also increasing evidence about the important role of dysregulation of the endogenous opioid system (59), the endocannabinoid system (1), or the nicotinic acetyl-choline receptor (nAChR) system (41) in the physiopathology of GD. Recent studies indicate that individuals with GD show a relative intolerance to pain that would involve the endogenous opioid system (59, 60). The dysfunction of the endogenous endocannabinoid system is known to be implicated in GD (61). The chronic activation of cannabinoid receptors (CB1) is associated with impairment of decision making (62). Although acute modulation by tetrahydrocannabinol has modest effects on decision making, it can play a substantial role in the regulation of the impulsive response. The nAChR system of the PFC is important to the decision-making process. An animal study with a mouse model lacking the nAChR system evidenced its crucial role for tuning of excitation and inhibition balance in the prelimbic cortex and hippocampus and for decision-making processes (41).

### Neurodevelopmental Perspective on Gambling Dual Disorder

From a neurobiological point of view, social activity early in life not only shapes the development of our brain but also induces reward, as suggested by evidence that the brain system that serves primary reward processing may also be critical for the processing of social relationships and attachment (1). The human brain develops slowly in a process that persists beyond the second decade of life and consists of multiple, organized, and highly dynamic steps, which are genetically determined, epigenetically directed, and environmentally influenced (63). According to categorical classifications of mental disorders, neurodevelopmental disorders are mental problems characterized by developmental deficits that present in childhood, such as autism spectrum disorder, intellectual disability, and ADHD (3). Nevertheless, brain changes underlie all mental disorders, starting early in development, potentially causing significant mental disorders, and dependent on other factors, including environmental ones. From this perspective, addictions and other mental disorders may be considered neurodevelopmental disorders. Therefore, a dual disorder can be detected in problems in childhood and adolescence. For many individuals, dual disorders start with the early expression of mental symptoms, such as impulsivity and oppositional behaviors, which in turn have been associated with dopaminergic system and BRS activity. These young, potentially vulnerable individuals with incomplete brain development engage in exploratory and impulsive behaviors which are preparing them for adulthood, but which may involve risks. If they choose to explore substances or gambling at this age they can develop a stronger response to such behaviors, leading to a dual disorder (47). Recent studies have found that immaturity of the frontal cortical and subcortical monoaminergic systems, which underlies adolescent impulsivity, can lead to increased vulnerability to addictive behaviors (e.g., GD) (64). On the other hand, patients with GD have shown an altered orbitofrontal sulcogyral pattern in both hemispheres compared with healthy controls (65). Addictive disorders associated with reward processing and decision making may be associated in part with neurodevelopmental disturbances of the orbitofrontal cortex and may be a possible transdiagnostic trait marker of early neurodevelopment in the social brain (66).

### Genetics of Gambling Dual Disorder

The role of genetics is essential in the development of human personality traits and clinical disorders. Different genetic polymorphisms lead to biological differences in brain circuits that support personality traits and make some individuals vulnerable to experiencing mental and behavioral disorders, while others are resilient (67). With the identification of more genetic variants relevant to addiction and related processes such as dual disorders, we may move into prevention and treatment in a truly individualized way (68). However, it is a long way from the genotype to phenotype, or how mental functions and disorders are ultimately expressed. This fact has driven the identification of intermediate steps, called endophenotypes. An endophenotype is a biological trait that is reliable in reflecting the function of a discrete biological system and is reasonably heritable, and as such, is more closely related to the root cause of the disease than the broad clinical phenotype (69, 70). Different personality traits have genetic and neurobiological differences (67, 71). Endophenotypes, such as personality traits, interact dynamically with the environment to ultimately determine the vulnerability or resilience of an individual to developing a dual disorder (67). Furthermore, GD can be observed more frequently in some families, and it is more commonly concordant in monozygotic than dizygotic twins (3). A family study in 31 individuals with GD and 31 healthy controls together with their first-degree relatives showed a significantly greater lifetime rate of GD among relatives of individuals with GD (8.3%) than in healthy controls (2.1%, odds ratio: 4.5) (72). Reliable data on heritability of GD will come from the Genome-Wide Association study (GWAS). However, for now, we have to refer to the analysis of 2 large registry studies (Vietnam Era Twin Registry and the Australian Twin Study of Gambling) involving 3,359 and 2,889 twin pairs, respectively (73, 74). Inherited factors could explain 35–54% of features associated with GD in the first registry and 40% in the second. The Australian Twin Registry also revealed sex differences in the heritability of gambling behaviors (75). The age of gambling onset was predominantly determined by genetic factors in men, but by shared environmental and genetic factors in women. Additionally, a meta-analysis of twin studies reported that GD is moderately heritable and moderately influenced by non-shared environmental factors (37). Furthermore, the magnitude of the genetic influence was greater in adults (53%) than adolescents (42%) and in males (47%) than females (28%). Likewise, diverse genetic studies have associated the role of dopaminergic (including D1, D2, and D4 dopamine receptor genes) and serotonergic genes (such as the DNA methylation of the serotonin transporter gene and monoamine oxidases A and B) with vulnerability to GD (76). Similarly, changes in DNA methylation of the dopamine receptor 2 gene (epigenetic modulation) have been correlated with treatment outcomes in GD, especially in individuals with high impulsivity (77). The genetics perspective is very useful for Gambling Dual Disorder and other behavioral addictions because it allows the identification of different specific phenotypes in different mental disorders. However, further research is needed to elucidate the specific genetic factors implicated in Gambling Dual Disorder vulnerability (2).

### Gambling Dual Disorder and Impulsivity

Despite the common belief that there is no such thing as an addictive personality, individuals with gambling addictions show personality traits (endophenotypes), such as sensation seeking and impulsivity. Impulsivity emerges from clinical evidence as the most important personality trait associated with GD and one that has an influence on the severity of the disorder (78). It may also be regarded as a vulnerability marker. Based on animal and human studies, impulsivity is familial and found in many categorical and dimensional psychiatric disorders (79). The construct of “impulsivity” has multiple cognitive and behavioral manifestations in daily life, similar to “impulsive lifestyle.” A recent meta-analysis revealed heightened impulsivity in GD and gambling problems (at risk individuals) across a range of cognitive domains (motor inhibition, attentional inhibition, discounting, and decision-making tasks), in keeping with neurobiological models (78). This data also demonstrated an elevated decision-making impulsivity even in those with less severe problem gambling; traits that are not routinely explored in this phenotype of gambling problem. Another study evaluating the influence of GD on decision making in connection with different impulsivity facets, reported increased impulsivity in nearly all analyzed dimensions in individuals with GD, compared with healthy controls, and a positive correlation between decision-making impairments and non-planning impulsivity only (80). Furthermore, deficient decision making was related to decreased gray matter volume in the medial orbitofrontal cortex.

Impulsive individuals are at risk of developing addiction to GD and also to stimulants (56). Problematic cocaine users were more likely to have GD in comparison to recreational users, and non-users. They also presented with increased levels of impulsivity trait, and other mental symptoms. These results emphasize the need for increased focus on dual disorders and treatment approaches specifically tailored to individuals with GD and cocaine addiction (81).

Nevertheless, it is important to note at this point that, from the perspective of precision psychiatry, addiction cannot be considered synonymous with impulsivity (7). There are cases of addictive dual disorder without significant impulsivity, with traits of negative emotionality, and symptoms of anxiety, dysphoria, and depression. Those play an important role in addiction to alcohol or opioids, whereas impulsivity is significant in the addiction to stimulants and behavioral additions such as Gambling Dual Disorder (82).

In considering trait impulsivity as an endophenotype, it is important to incorporate the drug of choice model and precision psychiatry concept. Due to the diversity in genotype and environment, “one men's meat is another man's poison” because the effects of substances and gambling are not the same among different individuals, including those of a different sex (7). The hypothesis is that, in spite of the similarities, there are also very important differences between addictions to different substances or gambling, and that these can be inferred from the dual disorders perspective. Findings support a “drug of choice model” and precision psychiatry concept, in which drugs from different drug classes do not produce common subjective responses in most individuals. Rather, individuals may be susceptible to particular drug classes (or gambling) based on personality traits or other individual neurobiological differences (6). For example, animal studies have confirmed that the impulsivity trait is able to predict compulsive consumption of cocaine, but not of heroin (32). From the point of view of diagnostic categories, impulsivity is a ubiquitous construct, and impulsivity is a core symptom of ADHD. In one study, current or lifetime ADHD prevalences in individuals with GD were 25.2% and 28.8% of the study population, respectively (83). These individuals with GD and ADHD had a higher prevalence of SUDs, personality disorders, and suicide attempts. Individuals with GD and ADHD spent more time gambling (83, 84). Gambling had a sedative (self-medication) effect on them, and they developed GD faster and more severely (85). It is interesting to note that this study considered only individuals with a full categorical diagnosis of ADHD. It did not consider a dimensional perspective, which would include individuals with less severe traits of ADHD, who may also develop gambling problems.

Another frequent diagnostic category of mental disorders in patients with Gambling Dual Disorder is antisocial personality disorder. Impulsivity is a possible but not mandatory symptom in the definition of antisocial disorder and psychopathy (9). In the population with GD, the Minnesota Multiphasic Personality Inventory shows an increase in the psychopathic deviate scale. Moreover, these individuals may also engage in antisocial acts and behaviors (86). Different mechanisms may be underlying in individuals with GD and antisocial personality disorder (some of them with distinct cognitive and neurobiological domains), challenging the unitary perspective of drug addiction and GD (87).

### Gambling Dual Disorder as Self-Medication

Similar to substance addiction, GD may be considered a type of self-medication. This psychodynamic perspective, proposed in the 1970s (88), has been reinforced by recent neurobiological research in animals and humans (32). Psychoactive substances have an impact on brain circuits and systems, such as the opioid, cannabinoid, and nicotinic systems, and ultimately on the dopaminergic BRS. These circuits and systems process emotions and perceptions that may have had a previous homeostatic imbalance (46). Children with impulsive behavior (with some type of ADHD) improved with the use of stimulants. Additionally, animal studies support the notion that impulsivity is normalized in impulsive rats with exposure to cocaine, providing some validity to self-medication hypothesis (44). This hypothesis is also applicable to behavioral addictions (83). Therefore, compulsive behaviors such as uncontrollable gambling, can be viewed as forms of self-medication from a dysfunctional dopaminergic BRS. The stimulation of dopaminergic BRS by compulsive gambling would act in a similar way to cocaine, in order to produce calm and relaxation.

Similarly, adolescents with ADHD are more prone to playing video games and thus, unconsciously, improve their attention deficit (89). Another example of GD as self-medication derives from the relationship between premenstrual symptoms and the associated perimenopausal depression in women (90). Women are at increased risk for gambling-related behaviors before and during menstruation (in comparison with other phases of the menstrual cycle) which may be a means of self-medication for the elevated negative affect (91).

### Gambling Dual Disorder and Sexual Differences

GD and other psychiatric disorders are frequently characterized by sexual differences in terms of prevalence, symptoms, and treatment response. Susceptibility, for instance, to depression, stress, and autism spectrum disorder is different in men and women, and it is related to genetic differences and brain circuits potentially involved in sexual dimorphism (92). From an evolutionary perspective, men and women show different attitudes and skills, experiencing different emotions in response to environmental and social stimuli, and also to stress, disease, and mental disorders (93). Women with GD usually report that they play because of stressful life situations or depressive states. By contrast, men do not associate gambling with emotional changes. GD presents earlier in men, although the progression is faster in women, starting in middle age (3). Among adolescents with GD, the proportion of males to females is ~3–5:1 (94). In a study with 996 high-school students, the occurrence of at-risk/problematic gambling was higher in males than females (24.8% vs. 2.9%) (95). There are also differences in game preferences. While men prefer strategic or action games, or the risk aspect of betting money, women usually choose non-strategic forms of gambling, which involve little (if any) personal decision making or skills, and where gamblers cannot influence or predict the outcome, such as lottery bingo or roulette (94, 96). Despite the fact that female adolescent gamblers have greater levels of psychological distress, male ones have enhanced impulsivity coping, sensation-seeking, and risk-taking behaviors (97). Males present higher levels of impulsivity than females, which could help to explain the prevalence of males in gambling engagement (98). Differences in brain circuits, number of receptors, receptor binding, and signaling together with hormonal influences can explain emotional and behavioral differences between males and females. Hormonal factors affecting women's gambling behavior are less well known. Investigations suggest that some addictive behaviors (including GD) can fluctuate over the menstrual cycle. Indeed, a recent study found that gambling behaviors (time spent gambling, money spent on gambling, and the probability of consuming alcohol while gambling) are exacerbated during ovulation (91).

## Conclusions

Gambling, an important behavior in the context of human evolution, is one that, from a dimensional perspective, can be dysfunctional, giving rise to problem gambling or, in severe cases, to GD. This state-of-the-art review highlights the difficulty of conceptualizing GD as a single nosological entity, defined only by gambling. Individuals with GD express a complex syndrome of multiple mental symptoms and different phenotypes that we are calling Gambling Dual Disorder. The existence of GD is related to neurobiological dysfunctions of the brain systems and circuits that are also involved in other mental disorders, i.e., dual disorders. The identification of the other mental disorders should not be restricted to DSM-5 diagnostic categories, but rather approached from a transdiagnostic perspective, including personality traits such as impulsivity. Here, we integrate accounts of the neurobiological mechanisms that underlie Gambling Dual Disorder from a transdiagnostic point of view, with overarching findings from clinical neuroscience and precision psychiatry to outline how gambling develops into addiction. We have also noted the importance of the “drug of choice” model, essential to the precision psychiatry perspective, in which we should identify specific phenotypes, endophenotypes and genetic markers that allow a personalized symptomatic treatment, not merely directed to the substance or the gambling behavior. Importantly, we emphasize the concept of Gambling Dual Disorder as a brain and neurodevelopmental disorder including the perspective of evolutionary psychiatry, genetics, impulsivity as an endophenotype, the self-medication hypothesis, and sexual biological differences. This broad vision of the disease advances a paradigm shift, highlighting how Gambling Dual Disorder should be conceptualized, diagnosed, and treated. For this reason, the re-conceptualization of GD as Gambling Dual Disorder, according to the perspective of clinical neuroscience and precision psychiatry, has become crucial.

## Author Contributions

All authors participated in the conception of the study. NS contributed to the writing of the manuscript, and CA supervised the review. All authors read, reviewed and approved the submitted version.

## Conflict of Interest

NS declares he has received honoraria for educational activities from Lundbeck, Indivior, Exeltis, and Shire. CA has been a consultant to or has received honoraria or grants from Acadia, Angelini, Gedeon Richter, Janssen Cilag, Lundbeck, Minerva, Otsuka, Roche, Sage, Servier, Shire, Schering Plough, Sumitomo Dainippon Pharma, Sunovion, and Takeda. The remaining authors declare that the research was conducted in the absence of any commercial or financial relationships that could be construed as a potential conflict of interest.
